# Supplementary material for: Genomic epidemiology of Delta SARS-CoV-2 during transition from elimination to suppression in Aotearoa New Zealand
Source: Nat Commun. 2022 Jul 12;13:4035. doi: 10.1038/s41467-022-31784-5 (PMC9274967; doi:10.1038/s41467-022-31784-5)

Supplementary Information. List of GISAID accession numbers and acknowledgements for global data used in Figure 1.

We gratefully acknowledge the following Authors from the Originating laboratories responsible for obtaining the specimens, as well as the Submitting laboratories where the genome data were generated and shared via GISAID, on which this research is based.

All Submitters of data may be contacted directly via www.gisaid.org Authors are sorted alphabetically.

EPI_ISL_3435684, EPI_ISL_3435695, EPI_ISL_3435699, EPI_ISL_3435705, EPI_ISL_3435708, EPI_ISL_3435714, EPI_ISL_3435725, EPI_ISL_3435752, EPI_ISL_3435753, EPI_ISL_3435770, EPI_ISL_3435772, EPI_ISL_3435777, EPI_ISL_3435778, EPI_ISL_3435781,
EPI_ISL_3435795, EPI_ISL_3435803, EPI_ISL_3435822, EPI_ISL_3435826, EPI_ISL_3435828, EPI_ISL_3435840, EPI_ISL_3435848, EPI_ISL_3435849, EPI_ISL_3435863, EPI_ISL_3435867, EPI_ISL_3435870, EPI_ISL_3435874, EPI_ISL_3435884, EPI_ISL_3435893,
EPI_ISL_3435894, EPI_ISL_3435900, EPI_ISL_3435903, EPI_ISL_3435908, EPI_ISL_3435924, EPI_ISL_3435927, EPI_ISL_3435937, EPI_ISL_3435951, EPI_ISL_3435956, EPI_ISL_3435960, EPI_ISL_3435962, EPI_ISL_3435966, EPI_ISL_3436811, EPI_ISL_3436847,
EPI_ISL_3436914, EPI_ISL_3436951, EPI_ISL_3436954, EPI_ISL_3436958, EPI_ISL_3437026, EPI_ISL_3437047, EPI_ISL_3437056, EPI_ISL_3437061, EPI_ISL_3437079, EPI_ISL_3437080, EPI_ISL_3437086, EPI_ISL_3437090, EPI_ISL_3437108, EPI_ISL_3437116,
EPI_ISL_3437126, EPI_ISL_3437133, EPI_ISL_3437152, EPI_ISL_3437167, EPI_ISL_3437175, EPI_ISL_3437191, EPI_ISL_3437217, EPI_ISL_3437239, EPI_ISL_3437240, EPI_ISL_3437256, EPI_ISL_3437266, EPI_ISL_3437267, EPI_ISL_3437278, EPI_ISL_3437295,
EPI_ISL_3437299, EPI_ISL_3437348, EPI_ISL_3437360, EPI_ISL_3437372, EPI_ISL_3437380, EPI_ISL_3437381, EPI_ISL_3437405, EPI_ISL_3437421, EPI_ISL_3437454, EPI_ISL_3437465

see above Berkshire and Surrey Pathology Services Wellcome Sanger Institute for the COVID-19 Genomics UK Berkshire and Surrey Pathology Services Lighthouse Laboratory and Alex Alderton; Cordelia Langford; David K. Jackson; Dominic Kwiatkowski; Ewan Harrison; Ian Johnston; Jeffrey Barrett; John Sillitoe on behalf of the Wellcome Sanger Lighthouse Laboratory (COG-UK) Consortium Institute COVID-19 Surveillance Team; Roberto Amato; Sonia Goncalves

EPI_ISL_3437049, EPI_ISL_3437059, EPI_ISL_3437064, EPI_ISL_3437094, EPI_ISL_3437103, EPI_ISL_3437124, EPI_ISL_3437144, EPI_ISL_3437149, EPI_ISL_3437202, EPI_ISL_3437204, EPI_ISL_3437212, EPI_ISL_3437219, EPI_ISL_3437223, EPI_ISL_3437244,
EPI_ISL_3437245, EPI_ISL_3437259, EPI_ISL_3437263

see above Health Services Laboratories Wellcome Sanger Institute for the COVID-19 Genomics UK Cordelia Langford; David K. Jackson; Dominic Kwiatkowski; Ewan Harrison; Health Services Laboratories and Alex Alderton; Ian Johnston; Jeffrey Barrett; John Sillitoe on behalf of the Wellcome Sanger Institute COVID-19 Surveillance Team; (COG-UK) Consortium Roberto Amato; Sonia Goncalves

EPI_ISL_3435464, EPI_ISL_3435465, EPI_ISL_3435466, EPI_ISL_3435471, EPI_ISL_3435489, EPI_ISL_3435502, EPI_ISL_3435503, EPI_ISL_3435515, EPI_ISL_3435517, EPI_ISL_3435523, EPI_ISL_3435531, EPI_ISL_3435541, EPI_ISL_3435558, EPI_ISL_3435563,
EPI_ISL_3435565, EPI_ISL_3435567, EPI_ISL_3435570, EPI_ISL_3435572, EPI_ISL_3435573, EPI_ISL_3435576, EPI_ISL_3435579, EPI_ISL_3435585, EPI_ISL_3435587, EPI_ISL_3435596, EPI_ISL_3435597, EPI_ISL_3435598, EPI_ISL_3435599, EPI_ISL_3435601,
EPI_ISL_3435616, EPI_ISL_3435618, EPI_ISL_3435628, EPI_ISL_3435635, EPI_ISL_3435651, EPI_ISL_3435659, EPI_ISL_3435661, EPI_ISL_3435698, EPI_ISL_3435730, EPI_ISL_3435744, EPI_ISL_3435748, EPI_ISL_3435775, EPI_ISL_3435800, EPI_ISL_3435817,
EPI_ISL_3435891, EPI_ISL_3435906, EPI_ISL_3435949, EPI_ISL_3435958, EPI_ISL_3435976, EPI_ISL_3435979, EPI_ISL_3435981, EPI_ISL_3435985, EPI_ISL_3435990, EPI_ISL_3435994, EPI_ISL_3436002, EPI_ISL_3436004, EPI_ISL_3436005, EPI_ISL_3436009,
EPI_ISL_3436010, EPI_ISL_3436015, EPI_ISL_3436017, EPI_ISL_3436019, EPI_ISL_3436024, EPI_ISL_3436025, EPI_ISL_3436028, EPI_ISL_3436038, EPI_ISL_3436039, EPI_ISL_3436041, EPI_ISL_3436042, EPI_ISL_3436048, EPI_ISL_3436049, EPI_ISL_3436053,
EPI_ISL_3436056, EPI_ISL_3436060, EPI_ISL_3436063, EPI_ISL_3436066, EPI_ISL_3436082, EPI_ISL_3436087, EPI_ISL_3436092, EPI_ISL_3436095, EPI_ISL_3436099, EPI_ISL_3436102, EPI_ISL_3436105, EPI_ISL_3436107, EPI_ISL_3436109, EPI_ISL_3436110,
EPI_ISL_3436111, EPI_ISL_3436115, EPI_ISL_3436121, EPI_ISL_3436125, EPI_ISL_3436126, EPI_ISL_3436133, EPI_ISL_3436135, EPI_ISL_3436139, EPI_ISL_3436144, EPI_ISL_3436148, EPI_ISL_3436150, EPI_ISL_3436154, EPI_ISL_3436156, EPI_ISL_3436159,
EPI_ISL_3436161, EPI_ISL_3436166, EPI_ISL_3436171, EPI_ISL_3436172, EPI_ISL_3436173, EPI_ISL_3436176, EPI_ISL_3436178, EPI_ISL_3436181, EPI_ISL_3436182, EPI_ISL_3436186, EPI_ISL_3436192, EPI_ISL_3436197, EPI_ISL_3436201, EPI_ISL_3436213,
EPI_ISL_3436217, EPI_ISL_3436220, EPI_ISL_3436221, EPI_ISL_3436224, EPI_ISL_3436227, EPI_ISL_3436229, EPI_ISL_3436230, EPI_ISL_3436246, EPI_ISL_3436247, EPI_ISL_3436249, EPI_ISL_3436251, EPI_ISL_3436252, EPI_ISL_3436256, EPI_ISL_3436259,
EPI_ISL_3436260, EPI_ISL_3436261, EPI_ISL_3436262, EPI_ISL_3436263, EPI_ISL_3436264, EPI_ISL_3436269, EPI_ISL_3436273, EPI_ISL_3436280, EPI_ISL_3436281, EPI_ISL_3436283, EPI_ISL_3436289, EPI_ISL_3436293, EPI_ISL_3436296, EPI_ISL_3436299,
EPI_ISL_3436300, EPI_ISL_3436308, EPI_ISL_3436314, EPI_ISL_3436319, EPI_ISL_3436321, EPI_ISL_3436322, EPI_ISL_3436323, EPI_ISL_3436324, EPI_ISL_3436325, EPI_ISL_3436334, EPI_ISL_3436335, EPI_ISL_3436336, EPI_ISL_3436343, EPI_ISL_3436348,
EPI_ISL_3436350, EPI_ISL_3436358, EPI_ISL_3436360, EPI_ISL_3436362, EPI_ISL_3436366, EPI_ISL_3436371, EPI_ISL_3436374, EPI_ISL_3436376, EPI_ISL_3436377, EPI_ISL_3436378, EPI_ISL_3436379, EPI_ISL_3436380, EPI_ISL_3436382, EPI_ISL_3436385,
EPI_ISL_3436386, EPI_ISL_3436387, EPI_ISL_3436391, EPI_ISL_3436395, EPI_ISL_3436400, EPI_ISL_3436403, EPI_ISL_3436412, EPI_ISL_3436413, EPI_ISL_3436417, EPI_ISL_3436422, EPI_ISL_3436423, EPI_ISL_3436428, EPI_ISL_3436431, EPI_ISL_3436436,
EPI_ISL_3436439, EPI_ISL_3436440, EPI_ISL_3436444, EPI_ISL_3436446, EPI_ISL_3436447, EPI_ISL_3436450, EPI_ISL_3436452, EPI_ISL_3436453, EPI_ISL_3436455, EPI_ISL_3436459, EPI_ISL_3436461, EPI_ISL_3436467, EPI_ISL_3436469, EPI_ISL_3436473,
EPI_ISL_3436474, EPI_ISL_3436476, EPI_ISL_3436479, EPI_ISL_3436485, EPI_ISL_3436497, EPI_ISL_3436502, EPI_ISL_3436503, EPI_ISL_3436506, EPI_ISL_3436511, EPI_ISL_3437492, EPI_ISL_3437493, EPI_ISL_3437494, EPI_ISL_3437498, EPI_ISL_3437504,
EPI_ISL_3437505, EPI_ISL_3437511, EPI_ISL_3437513, EPI_ISL_3437526, EPI_ISL_3437528, EPI_ISL_3437532, EPI_ISL_3437534, EPI_ISL_3437537, EPI_ISL_3437543, EPI_ISL_3437548, EPI_ISL_3437553, EPI_ISL_3437557, EPI_ISL_3437563, EPI_ISL_3437565,
EPI_ISL_3437567, EPI_ISL_3437571, EPI_ISL_3437572, EPI_ISL_3437580, EPI_ISL_3437589, EPI_ISL_3437591, EPI_ISL_3437593, EPI_ISL_3437594, EPI_ISL_3437600, EPI_ISL_3437603, EPI_ISL_3437611, EPI_ISL_3437612, EPI_ISL_3437616, EPI_ISL_3437618,
EPI_ISL_3437619, EPI_ISL_3437620, EPI_ISL_3437627, EPI_ISL_3437632, EPI_ISL_3437634, EPI_ISL_3437639, EPI_ISL_3437640, EPI_ISL_3437649, EPI_ISL_3437651, EPI_ISL_3437652, EPI_ISL_3437656, EPI_ISL_3437662, EPI_ISL_3437665, EPI_ISL_3437669,
EPI_ISL_3437673, EPI_ISL_3437674, EPI_ISL_3437680, EPI_ISL_3437688, EPI_ISL_3437692, EPI_ISL_3437703, EPI_ISL_3437712, EPI_ISL_3437716, EPI_ISL_3437717, EPI_ISL_3437724, EPI_ISL_3437727, EPI_ISL_3437732, EPI_ISL_3437745, EPI_ISL_3437747,
EPI_ISL_3437748, EPI_ISL_3437753, EPI_ISL_3437754, EPI_ISL_3437760, EPI_ISL_3437762, EPI_ISL_3437768, EPI_ISL_3437769, EPI_ISL_3437771, EPI_ISL_3437773, EPI_ISL_3437774, EPI_ISL_3437778, EPI_ISL_3437782, EPI_ISL_3437784, EPI_ISL_3437786,
EPI_ISL_3437790, EPI_ISL_3437793, EPI_ISL_3437794, EPI_ISL_3437800, EPI_ISL_3437801, EPI_ISL_3437802, EPI_ISL_3437807, EPI_ISL_3437814, EPI_ISL_3437815, EPI_ISL_3437820, EPI_ISL_3437821, EPI_ISL_3437823, EPI_ISL_3437827, EPI_ISL_3437831,
EPI_ISL_3437834, EPI_ISL_3437835, EPI_ISL_3437837, EPI_ISL_3437840, EPI_ISL_3437842, EPI_ISL_3437843, EPI_ISL_3437846, EPI_ISL_3437848, EPI_ISL_3437851, EPI_ISL_3437855, EPI_ISL_3437858, EPI_ISL_3437860, EPI_ISL_3437861, EPI_ISL_3437864,
EPI_ISL_3437867, EPI_ISL_3437871, EPI_ISL_3437874, EPI_ISL_3437875, EPI_ISL_3437876, EPI_ISL_3437877, EPI_ISL_3437879, EPI_ISL_3437884, EPI_ISL_3437886, EPI_ISL_3437887, EPI_ISL_3437891, EPI_ISL_3437893, EPI_ISL_3437897, EPI_ISL_3437898,
EPI_ISL_3437901, EPI_ISL_3437906, EPI_ISL_3437910, EPI_ISL_3437911, EPI_ISL_3437913, EPI_ISL_3437914, EPI_ISL_3437917, EPI_ISL_3437918, EPI_ISL_3437924, EPI_ISL_3437927, EPI_ISL_3437928, EPI_ISL_3437934, EPI_ISL_3437935, EPI_ISL_3437936,
EPI_ISL_3437941, EPI_ISL_3437947, EPI_ISL_3437949, EPI_ISL_3437950, EPI_ISL_3437953, EPI_ISL_3437956, EPI_ISL_3437958, EPI_ISL_3437959, EPI_ISL_3437961, EPI_ISL_3437963, EPI_ISL_3437964, EPI_ISL_3437966, EPI_ISL_3437968, EPI_ISL_3437969,
EPI_ISL_3437972, EPI_ISL_3437974, EPI_ISL_3437975, EPI_ISL_3437976, EPI_ISL_3437980, EPI_ISL_3437985, EPI_ISL_3437987, EPI_ISL_3437991, EPI_ISL_3437992, EPI_ISL_3437997, EPI_ISL_3437998, EPI_ISL_3437999, EPI_ISL_3438000, EPI_ISL_3438010,
EPI_ISL_3438012, EPI_ISL_3438015, EPI_ISL_3438017, EPI_ISL_3438021, EPI_ISL_3438023, EPI_ISL_3438027, EPI_ISL_3438028, EPI_ISL_3438029, EPI_ISL_3438033, EPI_ISL_3438036, EPI_ISL_3438037, EPI_ISL_3438039, EPI_ISL_3438041, EPI_ISL_3438043,
EPI_ISL_3438048, EPI_ISL_3438054, EPI_ISL_3438057, EPI_ISL_3438059, EPI_ISL_3438063, EPI_ISL_3438064, EPI_ISL_3438067, EPI_ISL_3438070, EPI_ISL_3438072, EPI_ISL_3438074, EPI_ISL_3438075, EPI_ISL_3438079, EPI_ISL_3438082, EPI_ISL_3438083,
EPI_ISL_3438085, EPI_ISL_3438087, EPI_ISL_3438090, EPI_ISL_3438092, EPI_ISL_3438093, EPI_ISL_3438096, EPI_ISL_3438099, EPI_ISL_3438104, EPI_ISL_3438107, EPI_ISL_3438109, EPI_ISL_3438110

see above Lighthouse Lab in Milton Keynes Wellcome Sanger Institute for the COVID-19 Genomics UK Cordelia Langford; David K. Jackson; Dominic Kwiatkowski; Ewan Harrison; Ian Johnston; Jeffrey Barrett; John Sillitoe on behalf of the Wellcome Sanger Institute COVID-19 Surveillance Team; Roberto Amato; Sonia Goncalves; The Lighthouse (COG-UK) Consortium Lab in Milton Keynes and Alex Alderton

EPI_ISL_3539339, EPI_ISL_3643439, EPI_ISL_3834019 4Cyte Pathology
EPI_ISL_3386969, EPI_ISL_3386972, EPI_ISL_3386973, EPI_ISL_3386975, EPI_ISL_3386976, EPI_ISL_3386977, EPI_ISL_3386979, EPI_ISL_3386980, EPI_ISL_3386981, EPI_ISL_3386982, EPI_ISL_3386983, EPI_ISL_3386986, EPI_ISL_3386987, EPI_ISL_3386988,
EPI_ISL_3386990, EPI_ISL_3386991, EPI_ISL_3386992, EPI_ISL_3386993, EPI_ISL_3386996

see above

EPI_ISL_4254544, EPI_ISL_4254545, EPI_ISL_4254548, EPI_ISL_4254551, EPI_ISL_4254554

EPI_ISL_3539334

EPI_ISL_3643668

AZDelta

Area of Virology, Serology and Virology Division (SAViD), New South Wales Health Pathology Randwick

Austech Medical Laboratories
Australian Clinical Labs (formerly Healthscope Pathology)

AZ Delta Medical Laboratories in Roeselare, Belgium

Virology Research Laboratory; Area of Virology, Serology and Virology Division (SAViD), New South Wales Health Pathology Randwick

NSW Health Pathology - Institute of Clinical Pathology and Medical Research; Westmead Hospital; University of Sydney

NSW Health Pathology - Institute of Clinical Pathology and Medical Research; Westmead Hospital; University of Sydney

Dieter De Smet; Frederik Van Hoecke; Geert Martens; on behalf of AZ Delta COVID-19 Genomics core (member of Genomic surveillance of SARS-CoV-2 in Belgium network) Au, J.; Bull, R.; Deveson, I.; Foster, C.; Rawlinson, W.; Ruiz Silva, M.; Van Hal, S.

Arnott A.; Draper J.; Gall M.; Martinez E.; Rockett R.; Sintchenko V.; on behalf of ICPMR

Arnott A.; Draper J.; Gall M.; Martinez E.; Rockett R.; Sintchenko V.; on behalf of ICPMR

Submitting Laboratory

Authors

NSW Health Pathology - Institute of Clinical Pathology and Medical Research; Westmead Hospital; University of Sydney

Arnott A.; Draper J.; Gall M.; Martinez E.; Rockett R.; Sintchenko V.; on behalf of ICPMR

EPI_ISL_3436560, EPI_ISL_3436609, EPI_ISL_3436625, EPI_ISL_3436688, EPI_ISL_3436702, EPI_ISL_3436781, EPI_ISL_3436790, EPI_ISL_3436820, EPI_ISL_3436828, EPI_ISL_3436829, EPI_ISL_3436839, EPI_ISL_3436860, EPI_ISL_3436869, EPI_ISL_3436872, EPI_ISL_3436874, EPI_ISL_3436878, EPI_ISL_3436891, EPI_ISL_3436960, EPI_ISL_3436977, EPI_ISL_3436980, EPI_ISL_3437000, EPI_ISL_3437024, EPI_ISL_3437042, EPI_ISL_3438111, EPI_ISL_3438114, EPI_ISL_3438116, EPI_ISL_3438118, EPI_ISL_3438119, EPI_ISL_3438123, EPI_ISL_3438128, EPI_ISL_3438129, EPI_ISL_3438130, EPI_ISL_3438133, EPI_ISL_3438136, EPI_ISL_3438139, EPI_ISL_3438142, EPI_ISL_3438143, EPI_ISL_3438145, EPI_ISL_3438149, EPI_ISL_3438151, EPI_ISL_3438153, EPI_ISL_3438154, EPI_ISL_3438155, EPI_ISL_3438156, EPI_ISL_3438157, EPI_ISL_3438159, EPI_ISL_3438160, EPI_ISL_3438163, EPI_ISL_3438167, EPI_ISL_3438169, EPI_ISL_3438172, EPI_ISL_3438173, EPI_ISL_3438402, EPI_ISL_3438414, EPI_ISL_3438435, EPI_ISL_3438441

see above Berkshire and Surrey Pathology Services Lighthouse Laboratory Wellcome Sanger Institute for the COVID-19 Genomics UK (COG-UK) Consortium Berkshire and Surrey Pathology Services Lighthouse Laboratory and Alex Alderton; Cordelia Langford; David K. Jackson; Dominic Kwiatkowski; Ewan Harrison; Ian Johnston; Jeffrey Barrett; John Sillitoe on behalf of the Wellcome Sanger Institute COVID-19 Surveillance Team; Roberto Amato; Sonia Goncalves

EPI_ISL_3446636, EPI_ISL_3446643 Cytogeneticka laborator Brno University Hospital Brno, CMBG

EPI_ISL_3417539, EPI_ISL_3417547, EPI_ISL_3417549, EPI_ISL_3417553, EPI_ISL_3417565, EPI_ISL_3417587, EPI_ISL_3417589, EPI_ISL_3417594, EPI_ISL_3417626, EPI_ISL_3417629, EPI_ISL_3417748, EPI_ISL_3417769, EPI_ISL_3417781, EPI_ISL_3417786, EPI_ISL_3417798, EPI_ISL_3417803, EPI_ISL_3417816, EPI_ISL_3417819, EPI_ISL_3417827, EPI_ISL_3417836, EPI_ISL_3417948, EPI_ISL_3417949, EPI_ISL_3417960, EPI_ISL_3417966, EPI_ISL_3417967, EPI_ISL_3417968, EPI_ISL_3417974, EPI_ISL_3417979, EPI_ISL_3418009, EPI_ISL_3418018, EPI_ISL_3418169, EPI_ISL_3418175, EPI_ISL_3418176, EPI_ISL_3418183, EPI_ISL_3418184, EPI_ISL_3418186, EPI_ISL_3418192, EPI_ISL_3418197, EPI_ISL_3418200, EPI_ISL_3418202, EPI_ISL_3418302, EPI_ISL_3418304, EPI_ISL_3418316, EPI_ISL_3418343, EPI_ISL_3418344, EPI_ISL_3418349, EPI_ISL_3418352, EPI_ISL_3418362, EPI_ISL_3418424, EPI_ISL_3418425, EPI_ISL_3418570, EPI_ISL_3418572, EPI_ISL_3418577, EPI_ISL_3418581, EPI_ISL_3418602, EPI_ISL_3418616, EPI_ISL_3418617, EPI_ISL_3418623, EPI_ISL_3418628, EPI_ISL_3418652

Bezdicek Matej; Dufkova Kristyna; Lengerova Martina; Svaton Jan; Volfova Pavlina

EPI_ISL_3417635, EPI_ISL_3417637, EPI_ISL_3417649, EPI_ISL_3417653, EPI_ISL_3417665, EPI_ISL_3417673, EPI_ISL_3417682, EPI_ISL_3417714, EPI_ISL_3417731, EPI_ISL_3417736, EPI_ISL_3417744, EPI_ISL_3417745, EPI_ISL_3417846, EPI_ISL_3417849, EPI_ISL_3417867, EPI_ISL_3417875, EPI_ISL_3417880, EPI_ISL_3417884, EPI_ISL_3417895, EPI_ISL_3417896, EPI_ISL_3417913, EPI_ISL_3417918, EPI_ISL_3417927, EPI_ISL_3417930, EPI_ISL_3418040, EPI_ISL_3418046, EPI_ISL_3418048, EPI_ISL_3418049, EPI_ISL_3418085, EPI_ISL_3418091, EPI_ISL_3418100, EPI_ISL_3418105, EPI_ISL_3418111, EPI_ISL_3418135, EPI_ISL_3418146, EPI_ISL_3418166, EPI_ISL_3418206, EPI_ISL_3418211, EPI_ISL_3418220, EPI_ISL_3418226, EPI_ISL_3418234, EPI_ISL_3418255, EPI_ISL_3418260, EPI_ISL_3418274, EPI_ISL_3418279, EPI_ISL_3418286, EPI_ISL_3418289, EPI_ISL_3418291, EPI_ISL_3418428, EPI_ISL_3418436, EPI_ISL_3418442, EPI_ISL_3418450, EPI_ISL_3418458, EPI_ISL_3418497, EPI_ISL_3418506, EPI_ISL_3418508, EPI_ISL_3418513, EPI_ISL_3418521, EPI_ISL_3418535, EPI_ISL_3418558,

see above Department of Bacteria, Parasites and Fungi, Statens Serum Statens Serum Institut Bioinformatics and Microbial Genomics Danish Covid-19 Genome Consortium Institut, Copenhagen, Denmark

EPI_ISL_3396463, EPI_ISL_3396469, EPI_ISL_3396528, EPI_ISL_3396531, EPI_ISL_3396533, EPI_ISL_3396536, EPI_ISL_3396541, EPI_ISL_3396543, EPI_ISL_3396546, EPI_ISL_3396551, EPI_ISL_3396554, EPI_ISL_3396556, EPI_ISL_3396559
see above Elisabeth Pharmacon University Hospital Brno, CMBG Jan Svaton; Kristyna Dufkova; Martina Lengerova; Matej Bezdicek; Pavlina Volfova

EPI_ISL_3426050, EPI_ISL_3426179, EPI_ISL_3426406, EPI_ISL_3426425, EPI_ISL_3539433, EPI_ISL_3539437, EPI_ISL_3539439, EPI_ISL_3539442, EPI_ISL_3643228, EPI_ISL_3643238, EPI_ISL_3643265, EPI_ISL_3643364, EPI_ISL_3643506, EPI_ISL_3643694, EPI_ISL_3643696

see above

EPI_ISL_3446654 EPI_ISL_3446606 EPI_ISL_3446645 EPI_ISL_3387257, EPI_ISL_3387258 EPI_ISL_3387254, EPI_ISL_3387255

see above Laverty Pathology NSW Health Pathology - Institute of Clinical Pathology and Medical Research; Westmead Hospital; University of Sydney

EPI_ISL_3719106, EPI_ISL_3722084, EPI_ISL_3722086, EPI_ISL_3722088, EPI_ISL_3800606, EPI_ISL_3800609, EPI_ISL_3800612

see above

EPI_ISL_3614809, EPI_ISL_3614812, EPI_ISL_3614814, EPI_ISL_3614816

EPI_ISL_3539330

New South Wales Health Pathology Royal Prince Alfred Hospital Queensland Health Forensic and Scientific Services

St Vincent's Pathology (SydPath)

Histopath

NSW Health Pathology - Institute of Clinical Pathology and Medical Research; Westmead Hospital; University of Sydney

Arnott A.; Draper J.; Gall M.; Martinez E.; Rockett R.; Sintchenko V.; on behalf of ICPMR

Bezdicek Matej; Dufkova Kristyna; Lengerova Martina; Svaton Jan; Volfova Pavlina Bezdicek Matej; Dufkova Kristyna; Lengerova Martina; Svaton Jan; Volfova Pavlina Bezdicek Matej; Dufkova Kristyna; Lengerova Martina; Svaton Jan; Volfova Pavlina Severine Berden et al. on behalf of the Jessa_cmdLab

Severine Berden et al. on behalf of the Jessa_cmdLab

Arnott A.; Draper J.; Gall M.; Martinez E.; Rockett R.; Sintchenko V.; on behalf of ICPMR

Hola Biolabs IFCOR Brno IFCOR Jihlava Jessa LKO

University Hospital Brno, CMBG University Hospital Brno, CMBG University Hospital Brno, CMBG Jessa

Jessa EPI_ISL_3643297, EPI_ISL_3643676, EPI_ISL_3643678, EPI_ISL_3643742, EPI_ISL_3707653, EPI_ISL_3707656, EPI_ISL_3707659, EPI_ISL_3707662, EPI_ISL_3707664, EPI_ISL_5778708,

EPI_ISL_5778709, EPI_ISL_5936283

EPI_ISL_3435461, EPI_ISL_3435467, EPI_ISL_3435469, EPI_ISL_3435478, EPI_ISL_3435488, EPI_ISL_3435497, EPI_ISL_3435498, EPI_ISL_3435510, EPI_ISL_3435532, EPI_ISL_3435554, EPI_ISL_3435564, EPI_ISL_3435600, EPI_ISL_3435608, EPI_ISL_3435622, EPI_ISL_3435636, EPI_ISL_3435638, EPI_ISL_3435640, EPI_ISL_3435646, EPI_ISL_3435649, EPI_ISL_3435652, EPI_ISL_3435672, EPI_ISL_3436284, EPI_ISL_3436285, EPI_ISL_3436286, EPI_ISL_3436297, EPI_ISL_3436310, EPI_ISL_3436320, EPI_ISL_3436330, EPI_ISL_3436331, EPI_ISL_3436332, EPI_ISL_3436346, EPI_ISL_3436373, EPI_ISL_3436390, EPI_ISL_3436406, EPI_ISL_3436421, EPI_ISL_3436432, EPI_ISL_3436438, EPI_ISL_3436449, EPI_ISL_3436454, EPI_ISL_3436460, EPI_ISL_3436504, EPI_ISL_3436507, EPI_ISL_3436508, EPI_ISL_3436520, EPI_ISL_3436522, EPI_ISL_3436523, EPI_ISL_3436524, EPI_ISL_3436529, EPI_ISL_3436551, EPI_ISL_3436568, EPI_ISL_3436572, EPI_ISL_3436573, EPI_ISL_3436577, EPI_ISL_3436585, EPI_ISL_3436594, EPI_ISL_3436600, EPI_ISL_3436605, EPI_ISL_3436614, EPI_ISL_3436616, EPI_ISL_3436619, EPI_ISL_3436626, EPI_ISL_3436636, EPI_ISL_3436639, EPI_ISL_3436643, EPI_ISL_3436648, EPI_ISL_3436649, EPI_ISL_3436660, EPI_ISL_3436672, EPI_ISL_3436674, EPI_ISL_3436680, EPI_ISL_3436683, EPI_ISL_3436687, EPI_ISL_3436689, EPI_ISL_3436698, EPI_ISL_3436700, EPI_ISL_3436710, EPI_ISL_3436713, EPI_ISL_3436715, EPI_ISL_3436717, EPI_ISL_3436723, EPI_ISL_3436729, EPI_ISL_3436730, EPI_ISL_3436736, EPI_ISL_3436738, EPI_ISL_3436739, EPI_ISL_3436743, EPI_ISL_3436751, EPI_ISL_3436754, EPI_ISL_3436766, EPI_ISL_3436774, EPI_ISL_3436778, EPI_ISL_3436792, EPI_ISL_3436801, EPI_ISL_3436804, EPI_ISL_3436808, EPI_ISL_3436812, EPI_ISL_3436817, EPI_ISL_3436833, EPI_ISL_3436845, EPI_ISL_3436846, EPI_ISL_3436850, EPI_ISL_3436861, EPI_ISL_3436862, EPI_ISL_3436879, EPI_ISL_3436881, EPI_ISL_3436893, EPI_ISL_3436896, EPI_ISL_3436909, EPI_ISL_3436912, EPI_ISL_3436915, EPI_ISL_3436917, EPI_ISL_3436931, EPI_ISL_3436935, EPI_ISL_3436945, EPI_ISL_3436946, EPI_ISL_3436952, EPI_ISL_3436974, EPI_ISL_3436978, EPI_ISL_3436979, EPI_ISL_3436982, EPI_ISL_3436983, EPI_ISL_3436990, EPI_ISL_3436993, EPI_ISL_3436997, EPI_ISL_3437008, EPI_ISL_3437023, EPI_ISL_3437029, EPI_ISL_3437031, EPI_ISL_3437032, EPI_ISL_3437037, EPI_ISL_3437060, EPI_ISL_3437065, EPI_ISL_3437096, EPI_ISL_3437102, EPI_ISL_3437160, EPI_ISL_3437190, EPI_ISL_3437194, EPI_ISL_3437197, EPI_ISL_3437214, EPI_ISL_3437218, EPI_ISL_3437222, EPI_ISL_3437237, EPI_ISL_3437248, EPI_ISL_3437257, EPI_ISL_3437260, EPI_ISL_3437286, EPI_ISL_3437290, EPI_ISL_3437293, EPI_ISL_3437303, EPI_ISL_3437309, EPI_ISL_3437310, EPI_ISL_3437316, EPI_ISL_3437331, EPI_ISL_3437333, EPI_ISL_3437335, EPI_ISL_3437337, EPI_ISL_3437339, EPI_ISL_3437346, EPI_ISL_3437351, EPI_ISL_3437358, EPI_ISL_3437364, EPI_ISL_3437367, EPI_ISL_3437368, EPI_ISL_3437373, EPI_ISL_3437385, EPI_ISL_3437390, EPI_ISL_3437413, EPI_ISL_3437433, EPI_ISL_3437440, EPI_ISL_3437446, EPI_ISL_3437447, EPI_ISL_3437450, EPI_ISL_3437458, EPI_ISL_3437460, EPI_ISL_3437464, EPI_ISL_3437478, EPI_ISL_3437487, EPI_ISL_3437488, EPI_ISL_3437491, EPI_ISL_3437496, EPI_ISL_3437525, EPI_ISL_3437541, EPI_ISL_3437559, EPI_ISL_3437574, EPI_ISL_3437584, EPI_ISL_3437597, EPI_ISL_3437614, EPI_ISL_3437623, EPI_ISL_3437630, EPI_ISL_3437637, EPI_ISL_3437647, EPI_ISL_3437648, EPI_ISL_3437678, EPI_ISL_3437683, EPI_ISL_3437694, EPI_ISL_3437701, EPI_ISL_3437705, EPI_ISL_3437718, EPI_ISL_3437734, EPI_ISL_3437736, EPI_ISL_3437737, EPI_ISL_3437749, EPI_ISL_3437751, EPI_ISL_3437770, EPI_ISL_3437776, EPI_ISL_3437796, EPI_ISL_3437797

see above

EPI_ISL_3643613

EPI_ISL_4882360, EPI_ISL_4882362, EPI_ISL_4882367

EPI_ISL_3419365, EPI_ISL_3419370 EPI_ISL_3418257, EPI_ISL_3418406

EPI_ISL_3446605 EPI_ISL_3446634, EPI_ISL_3446637 EPI_ISL_3446649

Lighthouse Lab in Alderley Park Medlab Pathology

Microbiological Diagnostic Unit - Public Health Laboratory (MDU- PHL)

Microbiology Department - University Hospital Brussel

Molekylær Medicinsk Afdeling, Aarhus University Hospital, Aarhus, Denmark

Nemocnice Breclav Nemocnice Jihlava Nemocnice Trebic

Wellcome Sanger Institute for the COVID-19 Genomics UK (COG-UK) Consortium

NSW Health Pathology - Institute of Clinical Pathology and Medical Research; Westmead Hospital; University of Sydney

Microbiological Diagnostic Unit - Public Health Laboratory (MDU-PHL)

Microbiology Department - University Hospital Brussel Statens Serum Institut Bioinformatics and Microbial Genomics

University Hospital Brno, CMBG University Hospital Brno, CMBG University Hospital Brno, CMBG

Microbiology RPAH
Queensland Health Forensic and Scientific Services

NSW Health Pathology - Institute of Clinical Pathology and Medical Research; Westmead Hospital; University of Sydney

Cordelia Langford; David K. Jackson; Dominic Kwiatkowski; Ewan Harrison; Ian Johnston; Jacquelyn Wynn; Jeffrey Barrett; John Sillitoe on behalf of the Wellcome Sanger Institute COVID-19 Surveillance Team; Mairead Hyland; Roberto Amato; Sonia Goncalves; The Lighthouse Lab in Alderley Park and Alex Alderton

Arnott A.; Draper J.; Gall M.; Martinez E.; Rockett R.; Sintchenko V.; on behalf of ICPMR Horan, K.; N.L.; Seemann, T.; Sherry

Oriane Soetens; Thomas Demuyser Danish Covid-19 Genome Consortium

Bezdicek Matej; Dufkova Kristyna; Lengerova Martina; Svaton Jan; Volfova Pavlina Bezdicek Matej; Dufkova Kristyna; Lengerova Martina; Svaton Jan; Volfova Pavlina Bezdicek Matej; Dufkova Kristyna; Lengerova Martina; Svaton Jan; Volfova Pavlina

Au, J.; Bull, R.; Deveson, I.; Foster, C.; Rawlinson, W.; Ruiz Silva, M.; Van Hal, S. Chenwei Wang on behalf of Q-PHIRE Genomics

Arnott A.; Draper J.; Gall M.; Martinez E.; Rockett R.; Sintchenko V.; on behalf of ICPMR Bezdicek Matej; Dufkova Kristyna; Lengerova Martina; Svaton Jan; Volfova Pavlina

Severine Berden et al. on behalf of the Jessa_cmdLab

St. Anne´s University Hospital Brno
see above ZOL Jessa

EPI_ISL_3446611, EPI_ISL_3446612
EPI_ISL_3387233, EPI_ISL_3387235, EPI_ISL_3387236, EPI_ISL_3387237, EPI_ISL_3387238, EPI_ISL_3387239, EPI_ISL_3387240, EPI_IS L_3387242, EPI_ISL_3387244, EPI_ISL_3387248, EPI_ISL_3387249, EPI_ISL_3387250, EPI_ISL_3387251

EPI_ISL_3418862, EPI_ISL_3418863 Baylor Scott & White-Temple Baylor Scott & White-Temple

Ari Rao; Kimberly Walker; Linden Morales; Marcus Volz; Shelby Johnson

EPI_ISL_3431119, EPI_ISL_3431131, EPI_ISL_3431139, EPI_ISL_3431141, EPI_ISL_3431162, EPI_ISL_3431166, EPI_ISL_3431190, EPI_IS L_3431194, EPI_ISL_3431198, EPI_ISL_3431209, EPI_ISL_3431239, EPI_ISL_3431240, EPI_ISL_3431255, EPI_ISL_3431259, EPI_ISL_3431268, EPI_ISL_3431269, EPI_ISL_3431295, EPI_ISL_3431306, EPI_ISL_3431307, EPI_ISL_3431319, EPI_ISL_3431327, EPI_ISL_3431336, EPI_ISL _3431342, EPI_ISL_3431358, EPI_ISL_3431367, EPI_ISL_3431388, EPI_ISL_3431398, EPI_ISL_3431405, EPI_ISL_3431410, EPI_ISL_3431413, EPI_ISL_3431427, EPI_ISL_3431448, EPI_ISL_3431473, EPI_ISL_3431513, EPI_ISL_3431517, EPI_ISL_3431563, EPI_ISL_3431564, EPI_ISL_ 3431567, EPI_ISL_3431584, EPI_ISL_3431594, EPI_ISL_3431597, EPI_ISL_3431601, EPI_ISL_3431605, EPI_ISL_3431627, EPI_ISL_3431636, EPI_ISL_3431645

see above

EPI_ISL_3398105, EPI_ISL_3398106, EPI_ISL_3398109, EPI_ISL_3398110

EPI_ISL_3386683 EPI_ISL_3386680 EPI_ISL_3386689 EPI_ISL_3394934, EPI_ISL_3394941 EPI_ISL_3411832, EPI_ISL_3411833

EPI_ISL_3432207 EPI_ISL_3419710

Broad Institute Clinical Research Sequencing Platform

CNR Institut Pasteur de la Guyane

Department of Public Health Botosani

Department of Public Health Iasi

Department of Public Health Suceava

Dept. of Microbiology and Infection Control, Akershus University Hospital HF

Hospital General Universitario de Ciudad Real Hospital Universitari Joan XXIII
Johor Bahru Public Health Laboratory

Infectious Disease Program, Broad Institute of Harvard and MIT Institut Pasteur de la Guyane

National Institute of Infectious Diseases-Prof. Dr. Matei Bals Molecular Diagnostics Laboratory

National Institute of Infectious Diseases-Prof. Dr. Matei Bals Molecular Diagnostics Laboratory

National Institute of Infectious Diseases-Prof. Dr. Matei Bals Molecular Diagnostics Laboratory

Dept. of Microbiology and Infection Control, Akershus University Hospital HF

Hospital General Universitario de Ciudad Real Hospital Universitari Joan XXIII

Institute for Medical Research, Infectious Disease Research Centre, National Institutes of Health, Ministry of Health Malaysia

Adams, G.; B.L.; B.W.; Bauer, M.; Birren; Blumenstiel, B.; Brown, C.; Carter, A.; Chaluvadi, S.; D.J.; DeFelice, M.; DeRuff, K.; Dodge, S.; Gabriel, S.; Gallagher, G.; Gladden-Young, A.; Granger, B.; J.E.; K.J.; Lagerborg, K.; Larkin, K.; Lee, M.; Lemieux; Lennon, N.; Loreth, C.; Madoff, L.; McGovern, S.; Meldrim, J.; Normandin, E.; P.C.; Park; Pearlman, L.; Reilly, S.; Rudy, M.; Sabeti; Siddle; Smole, S.; Tomkins-Tinch, C.; Vicente, G.; and MacInnis

Anne Lavergne; Antoine Enfissi; Arielle Salmier; Dominique Rousset

Corina Casangiu; Dan Otelea; Leontina Banica; Marius Surleac; Ovidiu Vlaicu; Petre Milu; Robert Hohan; Simona Paraschiv

Corina Casangiu; Dan Otelea; Leontina Banica; Marius Surleac; Ovidiu Vlaicu; Petre Milu; Robert Hohan; Simona Paraschiv

Corina Casangiu; Dan Otelea; Leontina Banica; Marius Surleac; Ovidiu Vlaicu; Petre Milu; Robert Hohan; Simona Paraschiv

Alexander Hesselberg Løvestad; Hege Vangstein Aamot

Cristina Colmenarejo; José Martínez-Alarcón; Lidia García-Agudo; Marta Torres-Narbona; Soledad Illescas Fernández-Bermejo Carla Martín; Clara Benavent; Cristina Gutiérrez; Ester Picó; Gemma Recio; Margarida Terrón; Natalia Bastón
Anasir MI; Azizan MA; Kamel K; Mohd Zawawi Z; Ramly N; Robert F; Suppiah J; Thayan R

EPI_ISL_3370277, EPI_ISL_3370282, EPI_ISL_3370283, EPI_ISL_3370285, EPI_ISL_3370292, EPI_ISL_3375606, EPI_ISL_3396589, EPI_ISL_3396617, EPI_ISL_3396633, EPI_ISL_3396700, EPI_ISL_3396713, EPI_ISL_3396728, EPI_ISL_3396753, EPI_ISL_3432292, EPI_ISL_3434692, EPI_ISL_3434709, EPI_ISL_3434711, EPI_ISL_3434712, EPI_ISL_3434713, EPI_ISL_3434714

see above Kansas Health and Environmental Lab Kansas Health and Environmental Lab

EPI_ISL_3436647 Lighthouse Lab in Alderley Park Wellcome Sanger Institute for the COVID-19 Genomics UK (COG- UK) Consortium

Amanda Bradley; Ben Olsen; Jonathan Barnell; Mike Grose; and Phil Adam

Cordelia Langford; David K. Jackson; Dominic Kwiatkowski; Ewan Harrison; Ian Johnston; Jacquelyn Wynn; Jeffrey Barrett; John Sillitoe on behalf of the Wellcome Sanger Institute COVID-19 Surveillance Team; Mairead Hyland; Roberto Amato; Sonia Goncalves; The Lighthouse Lab in Alderley Park and Alex Alderton

EPI_ISL_3435463, EPI_ISL_3435470, EPI_ISL_3435472, EPI_ISL_3435493, EPI_ISL_3435505, EPI_ISL_3435524, EPI_ISL_3435540, EPI_ISL_3435544, EPI_ISL_3435575, EPI_ISL_3435589, EPI_ISL_3435623, EPI_ISL_3435631, EPI_ISL_3435637, EPI_ISL_3435977, EPI_ISL_3435998, EPI_ISL_3436134, EPI_ISL_3436136, EPI_ISL_3436168, EPI_ISL_3436205, EPI_ISL_3436211, EPI_ISL_3436214, EPI_ISL_3436235, EPI_ISL_3436236, EPI_ISL_3436239, EPI_ISL_3436240, EPI_ISL_3436254, EPI_ISL_3436294, EPI_ISL_3436307, EPI_ISL_3436339, EPI_ISL_3436345, EPI_ISL_3436347, EPI_ISL_3436357, EPI_ISL_3436361, EPI_ISL_3436370, EPI_ISL_3436375, EPI_ISL_3436392, EPI_ISL_3436399, EPI_ISL_3436420, EPI_ISL_3436426, EPI_ISL_3436443, EPI_ISL_3436445, EPI_ISL_3436451, EPI_ISL_3436468, EPI_ISL_3436477, EPI_ISL_3436491, EPI_ISL_3436509, EPI_ISL_3436612, EPI_ISL_3436690, EPI_ISL_3436783, EPI_ISL_3436785, EPI_ISL_3436788, EPI_ISL_3436794, EPI_ISL_3436806, EPI_ISL_3436810, EPI_ISL_3436851, EPI_ISL_3436884, EPI_ISL_3436895, EPI_ISL_3436907, EPI_ISL_3436911, EPI_ISL_3436916, EPI_ISL_3436919, EPI_ISL_3436921, EPI_ISL_3436940, EPI_ISL_3436944, EPI_ISL_3436964, EPI_ISL_3436969, EPI_ISL_3436975, EPI_ISL_3436986, EPI_ISL_3436994, EPI_ISL_3437003, EPI_ISL_3437022, EPI_ISL_3437025, EPI_ISL_3437030, EPI_ISL_3437034, EPI_ISL_3437822, EPI_ISL_3437824, EPI_ISL_3437825, EPI_ISL_3437850, EPI_ISL_3437888, EPI_ISL_3437892, EPI_ISL_3437894, EPI_ISL_3437932, EPI_ISL_3437937, EPI_ISL_3437938, EPI_ISL_3437939, EPI_ISL_3437940, EPI_ISL_3437965, EPI_ISL_3438007, EPI_ISL_3438011, EPI_ISL_3438024, EPI_ISL_3438030, EPI_ISL_3438060, EPI_ISL_3438065, EPI_ISL_3438071, EPI_ISL_3438088, EPI_ISL_3438095, EPI_ISL_3438101, EPI_ISL_3438106, EPI_ISL_3438389, EPI_ISL_3438392, EPI_ISL_3438394, EPI_ISL_3438400, EPI_ISL_3438406, EPI_ISL_3438409, EPI_ISL_3438410, EPI_ISL_3438416, EPI_ISL_3438418, EPI_ISL_3438419, EPI_ISL_3438422, EPI_ISL_3438432, EPI_ISL_3438437, EPI_ISL_3438440, EPI_ISL_3438444, EPI_ISL_3438450, EPI_ISL_3438451, EPI_ISL_3438454

see above Lighthouse Lab in Glasgow Wellcome Sanger Institute for the COVID-19 Genomics UK (COG- Anna Dominiczak and Alex Alderton; Carol Clugston; Cordelia Langford; David Gray; David K. Jackson; Dominic Kwiatkowski; Ewan Harrison; Harper VanSteenhouse; Ian Johnston; Jeffrey Barrett; John Sillitoe on behalf of the UK) Consortium Wellcome Sanger Institute COVID-19 Surveillance Team; Roberto Amato; Sonia Goncalves; Yumi Kasai

EPI_ISL_3435687, EPI_ISL_3435690, EPI_ISL_3435732, EPI_ISL_3435734, EPI_ISL_3435751, EPI_ISL_3435760, EPI_ISL_3435783, EPI_ISL_3435788, EPI_ISL_3435796, EPI_ISL_3435813, EPI_ISL_3435833, EPI_ISL_3435841, EPI_ISL_3435842, EPI_ISL_3435846, EPI_ISL_3435854, EPI_ISL_3435871, EPI_ISL_3435872, EPI_ISL_3435885, EPI_ISL_3435909, EPI_ISL_3435916, EPI_ISL_3435930, EPI_ISL_3435941, EPI_ISL_3435945, EPI_ISL_3435955, EPI_ISL_3435961, EPI_ISL_3435967

see above Lighthouse Lab in Milton Keynes Wellcome Sanger Institute for the COVID-19 Genomics UK (COG- Cordelia Langford; David K. Jackson; Dominic Kwiatkowski; Ewan Harrison; Ian Johnston; Jeffrey Barrett; John Sillitoe on behalf of the Wellcome Sanger Institute COVID-19 Surveillance Team; Roberto Amato; Sonia Goncalves; The UK) Consortium Lighthouse Lab in Milton Keynes and Alex Alderton

EPI_ISL_3437058, EPI_ISL_3437069, EPI_ISL_3437077, EPI_ISL_3437093, EPI_ISL_3437101, EPI_ISL_3437104, EPI_ISL_3437110, EPI_ISL_3437127, EPI_ISL_3437130, EPI_ISL_3437143, EPI_ISL_3437145, EPI_ISL_3437146, EPI_ISL_3437148, EPI_ISL_3437158, EPI_ISL_3437161, EPI_ISL_3437162, EPI_ISL_3437170, EPI_ISL_3437173, EPI_ISL_3437176, EPI_ISL_3437186, EPI_ISL_3437192, EPI_ISL_3437193, EPI_ISL_3437199, EPI_ISL_3437207, EPI_ISL_3437210, EPI_ISL_3437227, EPI_ISL_3437231, EPI_ISL_3437233, EPI_ISL_3437235, EPI_ISL_3437236, EPI_ISL_3437251, EPI_ISL_3437258, EPI_ISL_3437261, EPI_ISL_3437262, EPI_ISL_3437275, EPI_ISL_3437283, EPI_ISL_3437284, EPI_ISL_3441976, EPI_ISL_3441984, EPI_ISL_3441987, EPI_ISL_3441991, EPI_ISL_3442006, EPI_ISL_3442017, EPI_ISL_3442055, EPI_ISL_3442084, EPI_ISL_3442089, EPI_ISL_3442112, EPI_ISL_3442151, EPI_ISL_3442158, EPI_ISL_3442163, EPI_ISL_3442170, EPI_ISL_3442174, EPI_ISL_3442176, EPI_ISL_3442182, EPI_ISL_3442184, EPI_ISL_3442208, EPI_ISL_3442211, EPI_ISL_3442216, EPI_ISL_3442225, EPI_ISL_3442231, EPI_ISL_3442251, EPI_ISL_3442257

see above

EPI_ISL_3425905
EPI_ISL_3426697, EPI_ISL_3426698, EPI_ISL_3426699

Lighthouse Laboratory Plymouth

Medica Microvida

Wellcome Sanger Institute for the COVID-19 Genomics UK (COG- UK) Consortium

Institute of Medical Virology Microvida

Cordelia Langford; David K. Jackson; Dominic Kwiatkowski; Ewan Harrison; Ian Johnston; Jeffrey Barrett; John Sillitoe on behalf of the Wellcome Sanger Institute COVID-19 Surveillance Team; Lighthouse Laboratory Plymouth and Alex Alderton; Roberto Amato; Sonia Goncalves

Alexandra Trkola; Annette Audigé; Cyril Shah; Gabriela Ziltener; Guido Bloemberg; Jon Huder; Jürg Böni; Kevin Steiner; Maria Grünberg; Maryam Zaheri; Michael Huber; Riccarda Capaul; Stefan Schmutz; Verena Kufner Jaco J. Verweij; Joep J. J. M. Stohr; Suzan D. Pas

EPI_ISL_3426927, EPI_ISL_3426928, EPI_ISL_3426929, EPI_ISL_3426930, EPI_ISL_3426932, EPI_ISL_3426933, EPI_ISL_3426934, EPI_ISL_3426935, EPI_ISL_3426951, EPI_ISL_3426952, EPI_ISL_3426953, EPI_ISL_3426954, EPI_ISL_3426955, EPI_ISL_3426956, EPI_ISL_3426957, EPI_ISL_3426958, EPI_ISL_3426974, EPI_ISL_3426975, EPI_ISL_3426976, EPI_ISL_3426977, EPI_ISL_3426979, EPI_ISL_3426980, EPI_ISL_3426981, EPI_ISL_3426983, EPI_ISL_3426999, EPI_ISL_3427000, EPI_ISL_3427001, EPI_ISL_3427002, EPI_ISL_3427003, EPI_ISL_3427004, EPI_ISL_3427005, EPI_ISL_3427007, EPI_ISL_3427026, EPI_ISL_3427027, EPI_ISL_3427028, EPI_ISL_3427030, EPI_ISL_3427031, EPI_ISL_3427033, EPI_ISL_3427034, EPI_ISL_3427037, EPI_ISL_3427052, EPI_ISL_3427053, EPI_ISL_3427054, EPI_ISL_3427055, EPI_ISL_3427056, EPI_ISL_3427057, EPI_ISL_3427058, EPI_ISL_3427059, EPI_ISL_3427077, EPI_ISL_3427078, EPI_ISL_3427079, EPI_ISL_3427090

EPI_ISL_3426936, EPI_ISL_3426937, EPI_ISL_3426938, EPI_ISL_3426939, EPI_ISL_3426940, EPI_ISL_3426941, EPI_ISL_3426942, EPI_ISL_3426943, EPI_ISL_3426944, EPI_ISL_3426945, EPI_ISL_3426946, EPI_ISL_3426947, EPI_ISL_3426948, EPI_ISL_3426949, EPI_ISL_3426959, EPI_ISL_3426960, EPI_ISL_3426961, EPI_ISL_3426962, EPI_ISL_3426963, EPI_ISL_3426964, EPI_ISL_3426965, EPI_ISL_3426966, EPI_ISL_3426967, EPI_ISL_3426968, EPI_ISL_3426969, EPI_ISL_3426970, EPI_ISL_3426972, EPI_ISL_3426973, EPI_ISL_3426984, EPI_ISL_3426985, EPI_ISL_3426986, EPI_ISL_3426987, EPI_ISL_3426988, EPI_ISL_3426989, EPI_ISL_3426990, EPI_ISL_3426991, EPI_ISL_3426992, EPI_ISL_3426994, EPI_ISL_3426995, EPI_ISL_3426996, EPI_ISL_3426997, EPI_ISL_3426998, EPI_ISL_3427008, EPI_ISL_3427010, EPI_ISL_3427011, EPI_ISL_3427012, EPI_ISL_3427013, EPI_ISL_3427014, EPI_ISL_3427015, EPI_ISL_3427016, EPI_ISL_3427017, EPI_ISL_3427018, EPI_ISL_3427019, EPI_ISL_3427020, EPI_ISL_3427021, EPI_ISL_3427023, EPI_ISL_3427038, EPI_ISL_3427039, EPI_ISL_3427040, EPI_ISL_3427041, EPI_ISL_3427042, EPI_ISL_3427043, EPI_ISL_3427044, EPI_ISL_3427045, EPI_ISL_3427046, EPI_ISL_3427047, EPI_ISL_3427048, EPI_ISL_3427049, EPI_ISL_3427050, EPI_ISL_3427051, EPI_ISL_3427060, EPI_ISL_3427061, EPI_ISL_3427062, EPI_ISL_3427063, EPI_ISL_3427064, EPI_ISL_3427065, EPI_ISL_3427067, EPI_ISL_3427068, EPI_ISL_3427069, EPI_ISL_3427070, EPI_ISL_3427071, EPI_ISL_3427073, EPI_ISL_3427075, EPI_ISL_3427076,

see above National Public Health Laboratory, National Centre for Infectious Diseases

National Public Health Laboratory, National Centre for Infectious Diseases

Katherine Ching; Lin Cui; Raymond Tzer Pin Lin; Royce Ang; Zhenyang Zhou

Allison Black; Brent Lee; Caitlin McDonnell; Eric Brandt; Erica Leasure; Glen McGillivary; Heather Blankenship; Holmes; Jade Mowery; Jennifer; Kelsey Florek; Keoni Omura; Kirtana Ramadugu; Quanta Brown; Stephanie Mccracken; Tyler Payne; and Tammy Bannerman

Sundqvist M et al

Alec Birchley; Alexander Adams; Amy Gaskin; Angela Marchbank; Bree Gatica-Wilcox; Catherine Moore; Jason Coombes; Joanne Watkins; Joel Southgate; Johnathan Evans; Laura Gifford; Lauren Gilbert; Lee Graham; Malorie Perry; Matthew Bull; Nicole Pacchiarini; Sally Corden; Sara Kumziene-Summerhayes; Sara Rey; Sarah Taylor; Simon Cottrell; Sophie Jones; Tom Connor

EPI_ISL_3298638 Ohio Department of Health Laboratory
EPI_ISL_3431683, EPI_ISL_3431685, EPI_ISL_3431686, EPI_ISL_3431687, EPI_ISL_3431688, EPI_ISL_3431689, EPI_ISL_3431721

see above

EPI_ISL_3422451, EPI_ISL_3422459, EPI_ISL_3422462, EPI_ISL_3422468, EPI_ISL_3422531, EPI_ISL_3422604

Orebro University Hospital, Dept Laboratory Medicine, Clinical Microbiology

Originating lab: Wales Specialist Virology Centre Sequencing lab: Pathogen Genomics Unit

Ohio Department of Health Laboratory

Orebro University Hospital

Public Health Wales Microbiology Cardiff Wales Specialist Virology Centre

EPI_ISL_3399079, EPI_ISL_3399080, EPI_ISL_3399081, EPI_ISL_3399082, EPI_ISL_3399083, EPI_ISL_3399084, EPI_ISL_3399085, EPI_ISL_3399086, EPI_ISL_3399087, EPI_ISL_3399088, EPI_ISL_3399089, EPI_ISL_3399090, EPI_ISL_3399091, EPI_ISL_3399092

see above Presidio Ospedaliero "Madonna delle Grazie" di ASM - P.O. Madonna delle Grazie - Matera Massimo Dell'Edera Matera

EPI_ISL_3425891, EPI_ISL_3425892 Stadtspital Waid Institute of Medical Virology Alexandra Trkola; Annette Audigé; Cyril Shah; Gabriela Ziltener; Guido Bloemberg; Jon Huder; Jürg Böni; Kevin Steiner; Maria Grünberg; Maryam Zaheri; Michael Huber; Riccarda Capaul; Stefan Schmutz; Verena Kufner

Supplementary Figure 1. A regular maximum likelihood phylogenetic tree shown in Figure 1C, showing the same sampling locations. Branches are scaled to the number of nucleotide substitutions per site.

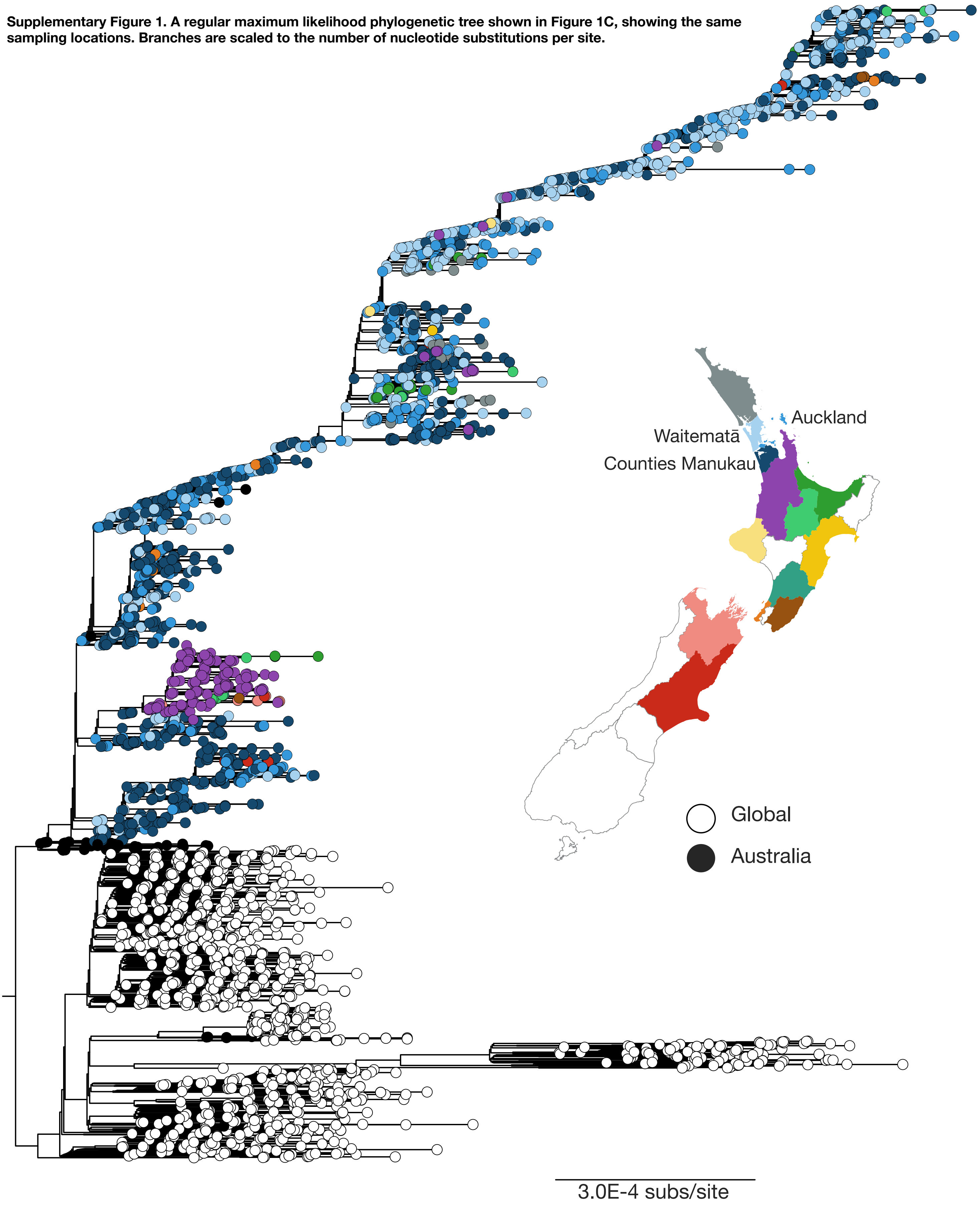

Supplementary Figure 2. Alignment of sequencing reads from a NZ isolate to the Wuhan hu-1 reference genome.

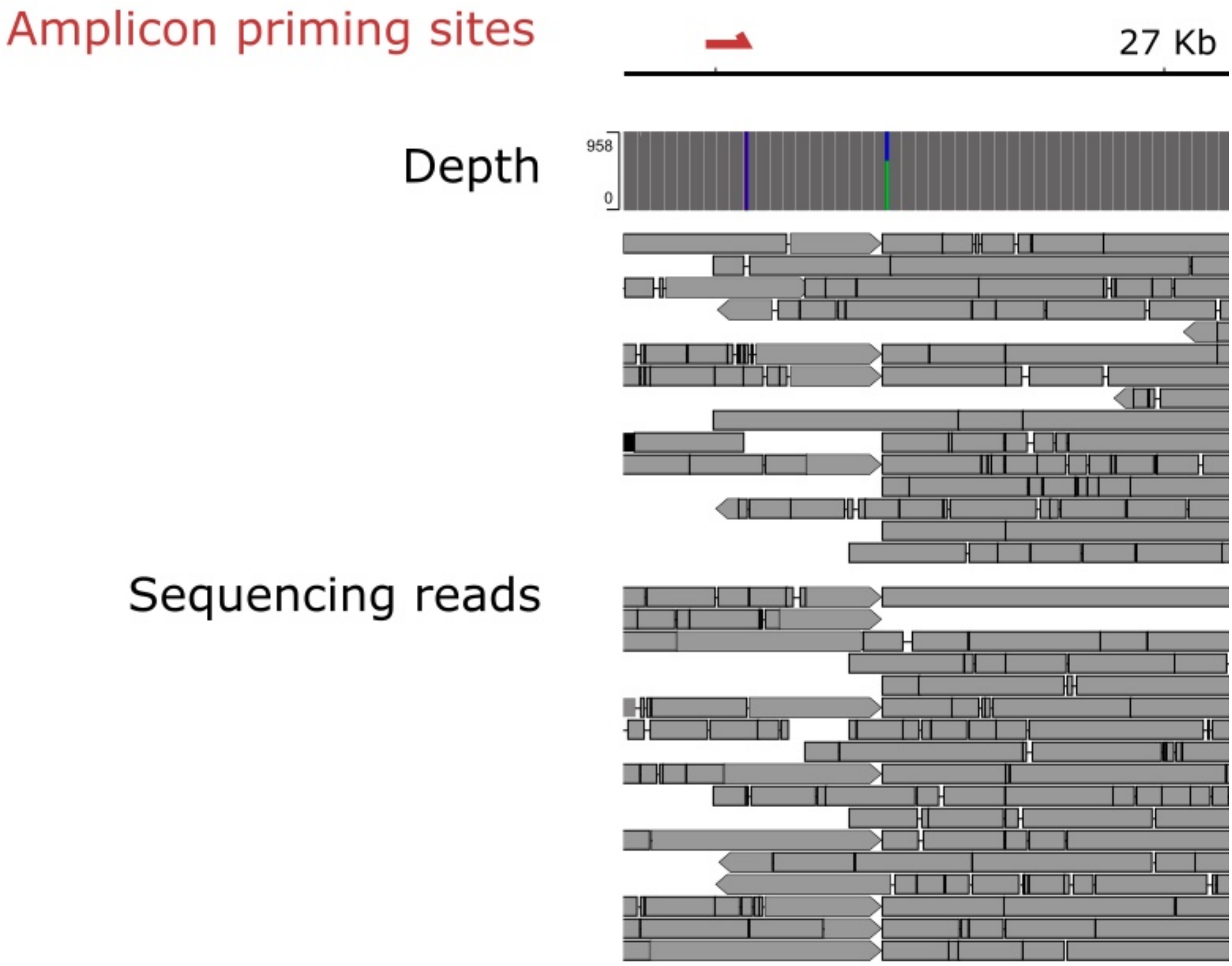

Supplement: Supplementary file 1 — Supplementary Information [file 41467_2022_31784_MOESM1_ESM.pdf]
