# Supplementary material for: Genomic epidemiology of Delta SARS-CoV-2 during transition from elimination to suppression in Aotearoa New Zealand
Source: Nat Commun. 2022 Jul 12;13:4035. doi: 10.1038/s41467-022-31784-5 (PMC9274967; doi:10.1038/s41467-022-31784-5)
Supplement: Supplementary file 3 — Description of Additional Supplementary Files [file 41467_2022_31784_MOESM3_ESM.pdf]

Description of Additional Supplementary Files

File Name: Supplementary Data 1
Description: GenBank accession numbers, patient age and sample location of genomes generated in this study.
