# Supplementary material for: Genomic epidemiology of Delta SARS-CoV-2 during transition from elimination to suppression in Aotearoa New Zealand
Source: Nat Commun. 2022 Jul 12;13:4035. doi: 10.1038/s41467-022-31784-5 (PMC9274967; doi:10.1038/s41467-022-31784-5)
Supplement: Supplementary file 5 — Reporting Summary [file 41467_2022_31784_MOESM5_ESM.pdf]

nature portfolio

Corresponding author(s): Jemma Geoghegan

Last updated by author(s): Jun 2, 2022

Reporting Summary

Nature Portfolio wishes to improve the reproducibility of the work that we publish. This form provides structure for consistency and transparency in reporting. For further information on Nature Portfolio policies, see our Editorial Policies and the Editorial Policy Checklist.

Statistics

For all statistical analyses, confirm that the following items are present in the figure legend, table legend, main text, or Methods section.

n/a	Confirmed	
☐	☒	The exact sample size (n) for each experimental group/condition, given as a discrete number and unit of measurement
☒	☐	A statement on whether measurements were taken from distinct samples or whether the same sample was measured repeatedly
☒	☐	The statistical test(s) used AND whether they are one- or two-sided<br>*Only common tests should be described solely by name; describe more complex techniques in the Methods section.*
☐	☒	A description of all covariates tested
☐	☒	A description of any assumptions or corrections, such as tests of normality and adjustment for multiple comparisons
☐	☒	A full description of the statistical parameters including central tendency (e.g. means) or other basic estimates (e.g. regression coefficient) AND variation (e.g. standard deviation) or associated estimates of uncertainty (e.g. confidence intervals)
☒	☐	For null hypothesis testing, the test statistic (e.g. F, t, r) with confidence intervals, effect sizes, degrees of freedom and P value noted<br>*Give P values as exact values whenever suitable.*
☐	☒	For Bayesian analysis, information on the choice of priors and Markov chain Monte Carlo settings
☐	☒	For hierarchical and complex designs, identification of the appropriate level for tests and full reporting of outcomes
☒	☐	Estimates of effect sizes (e.g. Cohen's d, Pearson's r), indicating how they were calculated

Our web collection on statistics for biologists contains articles on many of the points above.

Software and code

Policy information about availability of computer code

Data collection	Data are available from GenBank (accession numbers: ON669306-ON692908). No software was used for data collection.
Data analysis	We used open source software for this study: ARTIC bioinformatics medaka pipeline (v 1.1.0), MAFFT (v 7), IQ-TREE (v 1.6.8), PANGO (v 2.0) (https://github.com/hCoV-2019/pangolin), BEAST (v 2.5), Matlab (2020b).

For manuscripts utilizing custom algorithms or software that are central to the research but not yet described in published literature, software must be made available to editors and reviewers. We strongly encourage code deposition in a community repository (e.g. GitHub). See the Nature Portfolio guidelines for submitting code & software for further information.

Data

Policy information about availability of data

All manuscripts must include a data availability statement. This statement should provide the following information, where applicable:
- Accession codes, unique identifiers, or web links for publicly available datasets
- A description of any restrictions on data availability
- For clinical datasets or third party data, please ensure that the statement adheres to our policy

Genomic data has been submitted to GenBank under accession numbers: ON669306-ON692908.

Field-specific reporting

Please select the one below that is the best fit for your research. If you are not sure, read the appropriate sections before making your selection.

[x] Life sciences [ ] Behavioural & social sciences [ ] Ecological, evolutionary & environmental sciences

For a reference copy of the document with all sections, see nature.com/documents/nr-reporting-summary-flat.pdf

Life sciences study design

All studies must disclose on these points even when the disclosure is negative.

Sample size	There was a total of 8974 laboratory-confirmed cases of COVID-19 in New Zealand during the study period. A total of 3806 laboratory-confirmed samples of SARS-CoV-2 were received by the authors for whole genome sequencing and these generated genomes that passed quality control, which were used in this study. No sample size calculation was performed. Instead, we generated genomes from all samples received.
Data exclusions	Nucleotide positions that were less than 20x coverage were masked to N in the final consensus genome. Positions with an alternative allele frequency between 20% to 79% were also masked to N. Genomes that contained more than 10% of Ns did not pass quality control and were excluded from the analysis. This exclusion criteria was pre-established.
Replication	All phylogenetic trees and statistical tests were run in duplicate by multiple authors, namely J.L. Geoghegan and J. Douglas and were successful.
Randomization	Our analysis included 1500 uniformly sampled at random global SARS-CoV-2 genomes. This was only to show on a phylogenetic tree (Figure 1) and these data were not included in any of the formal analysis.
Blinding	Blinding was not relevant to this study since data included de-identified genomes and therefore is not applicable.

Reporting for specific materials, systems and methods

We require information from authors about some types of materials, experimental systems and methods used in many studies. Here, indicate whether each material, system or method listed is relevant to your study. If you are not sure if a list item applies to your research, read the appropriate section before selecting a response.

Materials & experimental systems

n/a	Involved in the study	
[x]	[ ]	Antibodies
[x]	[ ]	Eukaryotic cell lines
[x]	[ ]	Palaeontology and archaeology
[x]	[ ]	Animals and other organisms
[x]	[ ]	Human research participants
[x]	[ ]	Clinical data
[x]	[ ]	Dual use research of concern

Methods

n/a	Involved in the study	
[x]	[ ]	ChIP-seq
[x]	[ ]	Flow cytometry
[x]	[ ]	MRI-based neuroimaging
